# Road traffic accidental injuries and deaths: A neglected global health issue

**DOI:** 10.1002/hsr2.1240

**Published:** 2023-05-02

**Authors:** Sirwan K. Ahmed, Mona G. Mohammed, Salar O. Abdulqadir, Rabab G. Abd El‐Kader, Nahed A. El‐Shall, Deepak Chandran, Mohammad E. Ur Rehman, Kuldeep Dhama

**Affiliations:** ^1^ Department of Pediatrics Rania Pediatric & Maternity Teaching Hospital Rania Iraq; ^2^ Department of Nursing University of Raparin Rania Iraq; ^3^ RAK College of Nursing RAK Medical and Health Sciences University Ras Al Khaimah UAE; ^4^ Faculty of Nursing Mansoura University Mansoura Egypt; ^5^ Department of Poultry and Fish Diseases, Faculty of Veterinary Medicine Alexandria University Edfina Egypt; ^6^ Department of Veterinary Sciences and Animal Husbandry, Amrita School of Agricultural Sciences Amrita Vishwa Vidyapeetham University Coimbatore Tamil Nadu India; ^7^ Department of Medicine Rawalpindi Medical University Rawalpindi Pakistan; ^8^ Division of Pathology ICAR‐Indian Veterinary Research Institute Bareilly Uttar Pradesh India

**Keywords:** autonomous vehicles, death, global health, management, prevention, risk factors, road traffic accidents

## Abstract

Across the world, traffic accidents cause major health problems and are of concern to health institutions; nearly 1.35 million people are killed or disabled in traffic accidents every year. In 2019, 93% of road traffic injury‐related mortality occurred in low‐ and middle‐income countries with an estimated burden of 1.3 million deaths. This issue is growing; by 2030, road traffic injuries will be the seventh leading cause of death globally. The present report highlights an overview of road traffic accidents, accidental injuries, and deaths, associated risk factors, important precautions, safety rules, and counteracting management strategies. In modern cultures, road accidents are a major source of death and serious injuries. Road traffic injuries are a substantial yet underserved public health issue around the world that requires immediate attention. To prevent accidents in the long term, it is essential to adopt conservative preventive measures that can minimize collisions and promote a safe road environment.

## 1 INTRODUCTION

Across the world, traffic accidents cause majorhealth problems and are of concern to health institutions, nearly 1.35 million people are killed or disabled in traffic accidents every year, about 3700 people die every day in fatal accidents alone, half of which are cyclists, motorcyclists, or pedestrians, as vulnerable road users.^1^ For example, in the United States, car accidents are considered one of the main causes of death. In 2020 alone, about 40,000 people died in a traffic accident and about 2.1 million people visited emergency units due to traffic accidents. That is estimated at $430 billion in medical costs and quality of life and lives lost.^2^

Traffic injuries are the leading cause of death among children and youth aged 5–29, with 20 to 50 million injuries that do not cause death but cause disability and serious economic damage to individuals, families, and countries; the loss is due to the cost of medical care and lost wages of the infected or deceased person. Their families are often forced to leave work or school to care for their injured in traffic accidents. According to the WHO, the cost of road traffic crashes in most countries is estimated to be around 3% of their GDP.^3^ The present report highlights an overview of road traffic accidents, accidental injuries, and deaths, associated risk factors, important precautions, safety rules, and counteracting management strategies.

## 2 CONCEPT DEFINITIONS

An accident is defined as an unexpected and uncontrollable incident in which the action and reaction of an item or person causes personal injury or property damage. A traffic accident is defined as the failure of the road vehicle driver system to perform one or more activities required for the trip to be completed without harm or loss. Traffic accidents are mostly caused by poor maintenance of the road network and a lack of efficient and systematic enforcement.^4^

The main contributors to traffic accidents include poor road conditions, reckless passing, drowsy driving, sleepwalking, intoxication, illness, use of mobile phones, eating and drinking in the car, inattention in the event of a street accident, and the inability of other drivers to react quickly enough to the situation.^5^, ^6^ Regardless of the vehicle type, traumatic injuries after traffic accidents can affect any part of the body. Vulnerable parts of the body that lead to fatal consequences include the head, chest, abdominal pelvis, and spine.^7^

The general health and well‐being of citizens in the United States depend on safe transportation. It is a crucial component of the country's transportation and travel infrastructure.^8^ Safety breaches occur through harms caused by inadvertent behaviors or events. The main objective of transportation safety planning is to increase safety by supporting initiatives to create policies, programs, and projects related to all transportation infrastructure, and it also aims to reduce the number of injuries and deaths caused by traffic accidents on public roads.^9^ Establishing a strong transportation policy will have a direct impact on individual health, such as reducing exposure to air pollution and the problems caused by air pollution. Furthermore, it is the right of everyone to have access to safe, healthy, and affordable transportation. The government then prioritizes supporting a healthy society with innovative and modern designs and providing appropriate and safe opportunities by building a safe and an appropriate transportation infrastructure that can reduce the rate of injuries and deaths from transportation accidents is a priority of the government.^10^

## 3 CAUSES AND RISK FACTORS

Regarding risk factors for traffic accidents, socioeconomic status is considered the main cause of traffic accidents. According to WHO data, road traffic accidents disproportionately affect low‐ and middle‐income countries, where 90% of all road traffic deaths occur despite these countries having only 60% of the world's vehicles, African countries have higher rates of traffic accidents than European countries, which have lower rates of traffic accidents, even in high‐income countries, people with poor economic conditions are more likely to be involved in traffic accidents.^3^ Traffic and road accidents are considered one of the leading human causes of death among children and adolescents aged 5–29 years. Another reason is sex; men are more likely to be involved in traffic accidents than women, especially young men under the age of 25, hey accounting for 3 out of 4 deaths in traffic accidents (73%), which is three times more likely than their peer girls. However, the greatest causes of death in traffic accidents are not wearing seat belts, restraining children, and not wearing helmets, which certainly reduces the risk of brain injury and death in motorcycle accidents, the risk of injury and death to the back seat and front passengers is also related to the use of seatbelts.^3^

In general, the approach to the road safety system approach is more based on the risk‐based method, considering human mistakes and trying to ensure the safety of road users. In general, the approach to the road safety system approach is more based on the risk‐based method, considering human errors, and trying to ensure the safety of road users. The system can be designed to be forgiving of human errors and consider human sensitivity toward traffic victims, including; safety roads, roadside safety, safety speed, safety vehicles and safe users, and all these principles must be considered to reduce the number of victims in traffic accidents.^3^

There is a direct relationship between speeds and the possibility occurring of an accident, as well as severity of the events; with a 1% increase in mean speeds increases the probability occurring of the fatal crash by 4% and an increase in the risk of serious injuries by 3%. The use of alcohol and other substances while driving increases the risk of fatal accidents, resulting in serious deaths or injuries. If a driver's test of alcohol is more than 0.04 grams per deciliter (G/DL), the risk of traffic accidents is very high. Then the type of drug used by drivers will change the result of traffic accidents, for example, those who have used a stimulant drug such as amphetamines are five times more likely to have a fatal accident than those who did not take the drug.^3^

Another cause of traffic accidents is being busy while driving, which can cause serious damage. Mobile operation is a crucial issue for driving safety that requires intervention. For example, when people are busy with their mobile phones while driving, the risk of traffic accidents increases about four times more than those who do not use their mobile phones while driving. The reason is that the use of mobile phones while driving disrupts the person's reaction to the brakes and traffic signs and makes it unable to stay on the line and follow the appropriate distance. What is expected to increase day by day increases the risk of crash with mobile phone use.^3^

Furthermore, road design has a significant impact on road safety. This covers the safety of all road users, for example, pedestrians, cyclists, and motorcyclists. It is very important to consider the safety of all road users when designing roads. To reduce the risk of accidents for road users, streets, cycle lanes, safe crossings, and other traffic calming measures are very important. Then, unsafe vehicles that are not of good quality or do not meet fundamental norms of vehicle safety, because, of course, vehicle safety is very important to prevent traffic accidents and reduce the risk of accidents. According to available data, the risk of traffic accidents for vehicle occupants and pedestrians increases significantly in the absence of fundamental norms of vehicle safety requirements.^3^

## 4 MANAGEMENT

Medical management it is very important to familiarize drivers and people that in the event of a road accident, the first step is to call an ambulance and road health centers immediately and go to a nearby hospital. Medication can aid in healing if the injury is not too severe. The administration of tetanus shots and pain relievers by injection is an example of common safety precautions.^11^ In the event of severe blood loss, a blood transfusion may be necessary. In case of bone fractures, doctors do very well and scientifically place the bones in place, and the replacement of a bone that has been dislocated, but does not need surgery is called a closed reduction. In extreme circumstances, surgical intervention is required to operate on the victims of fatal accidents in emergency to save their lives. The surgeon also should be aware of the hidden injuries beneath a seemingly healthy abdomen and other parts of the body. Blunt organ trauma, as well as penetrating spine and back trauma, are all possibilities.^12^, ^13^

However, delays in identifying and treating people injured in a road traffic accident worsen their conditions. In addition, secure the treatment of pre‐hospital for the injured in traffic accidents as soon as possible and then receive the necessary treatment in the hospital is to increase the standard of health care for postcrash care and is considered one of the necessary tasks.

The implementation of traffic laws by drivers, including the law on drinking during driving, wearing safety belts, complying with speed limits, wearing helmets, and stabilizing children strictly in private places, reducing the death rate and injuries related to these special behaviors.^3^

Injuries and fatalities from motor vehicle collisions can be avoided by following appropriate precautions and safety rules. Understanding the dangers and taking precautions to ensure health and safety while driving, at home or abroad, can help prevent these accidents and deaths. Always buckle up, regardless of how short the trip of your trip/travel is a car passenger whether sit in front or back seat of the car, should be sure he is tied with belt seats, and children should be attached in the back of the car on a special seat that is suitable for their age, height, and weight. When operating a motorcycle, motorbike, or bicycle, always wear a helmet. Avoid traveling with a drunk driver and never drive while drunk or high. Speed limits must be respected and drive without interruptions or distractions. For example, refrain from texting, emailing, or accessing social networks while driving. Crossing the street should always be done with caution, especially in countries where the steering wheel is on the left side of the car. Only use cabs that are clearly identified and try to choose seatbelt‐equipped taxis. Avoid traveling in large, heavy, or packed minivans or buses that are top‐heavy, overweight, or packed.^14^, ^15^

It is the duty of governments to work to provide the conditions of road safety, which is in coordination with different sectors such as transportation, health, law, education, and civilian organizations that work to raise awareness of society. Then design a safe traffic infrastructure, stabilize equipment and signs roads, improve the quality of vehicles and other means of transportation, monitor traffic accidents for victims and repair roads, implement and establish traffic laws related to traffic dangers, expand transportation trails, conduct scientific research, raise awareness of society with traffic laws and using the means of transportation.^3^, ^10^

Existing interventions in high‐income countries aimed at reducing road traffic accidents and injuries have shown to be effective.^15^ Speed cameras, seatbelt laws, and campaigns have all been successful in reducing the number of accidents and injuries on the roads.^15^

Collecting and monitoring data on road traffic accidents and injuries is crucial for understanding trends and identifying areas where improvements can be made. Data can provide insight into the causes of accidents and injuries, which can help inform interventions and policies. Types of data needed include the number and severity of accidents, the demographics of those involved, and the types of vehicles and infrastructure involved.^16^ Challenges in collecting and analyzing data include inconsistencies in reporting, limited resources for data collection and analysis, and privacy concerns.^17^

Government agencies have a significant role to play in setting road safety regulations and standards, as well as providing funding for infrastructure improvements and education and training programs. Vehicle manufacturers can also contribute by designing and producing safer vehicles with advanced safety features. Road safety organizations can raise awareness and promote education and training programs for drivers, pedestrians, and cyclists.

Emerging technologies, such as autonomous vehicles, have the potential to greatly reduce the number of accidents caused by human error. However, this technology is still in its early stages, and there are many challenges that need to be addressed before it can be fully implemented. Implications for road safety policies and programs include creating new safety regulations, providing training for law enforcement officers and emergency responders on how to deal with autonomous vehicles, and ensuring that infrastructure is designed to accommodate these new technologies.

**Figure 1:**
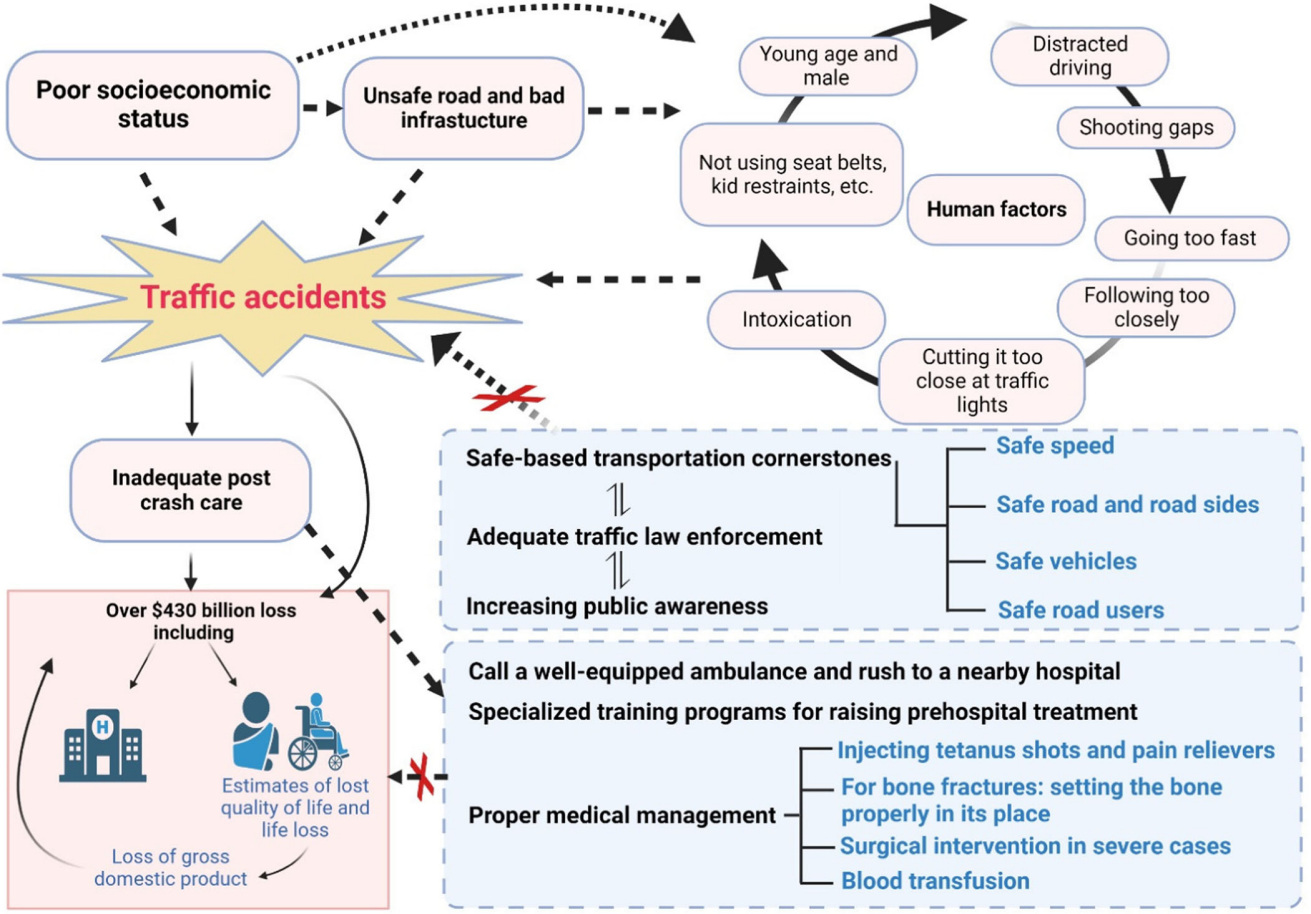
A pictorial representation on road traffic accidental injuries and deaths, risk factors, salient precautions, safety rules, and management strategies. Figure was designed by Biorender.com program.

An overview of accident and death injuries and deaths in road traffic, associated risk factors, salient precautions, safety rules, and counteracting management strategies to be adopted is shown in Figure 1.

To reduce traffic accidents and the damage caused by traffic accidents, we can rely on raising awareness among people in general and drivers in particular. Also, orient them about the risks, safety measures, and necessary preventive measures during driving. In general, it can be said that public awareness, road safety infrastructure, and traffic rules are the most effective reasons for reducing traffic accidents. Transportation is not only important in contemporary life, but has also become crucial and resourceful.

Raising public awareness and overcoming barriers in low‐income countries to reduce traffic accidents, injuries, and deaths can be achieved through several strategies (Table 1).

**Table 1:** Strategies to reduce traffic accidents.

1	Education and Training	One of the most effective ways to raise public awareness about road safety is through education and training. Governments, NGOs, and other organizations can conduct training sessions for drivers, pedestrians, and other road users to teach them about safe driving practices, traffic rules, and pedestrian safety.
2	Campaigns	Awareness campaigns can be launched through various mediums, such as billboards, radio, TV, social media, and other platforms to reach a wider audience. These campaigns can highlight the importance of road safety and the consequences of reckless driving.
3	Infrastructure Development	Improving infrastructure such as road signage, pedestrian crossings, and street lighting can help in reducing accidents. Governments can also construct speed bumps, roundabouts, and other traffic‐calming measures to reduce speeding and improve road safety.
4	Law Enforcement	Effective law enforcement can deter reckless driving and encourage adherence to traffic rules. Governments can increase the number of traffic police and use technology such as speed cameras to catch violators and enforce penalties.
5	Partnerships	Partnerships between governments, NGOs, and other organizations can help to overcome barriers in low‐income countries. For example, NGOs can provide funding and expertise to governments for road safety programs.
6	Community Engagement	Community engagement is important to create a sense of ownership and responsibility towards road safety. Local organizations can conduct awareness campaigns and engage with communities to encourage safe driving practices and promote pedestrian safety.

## 5 CONCLUSION

Increased traffic input is unavoidable; however, the outcome must be taken carefully in terms of producing accidents. It should be noted that the increase in accidents requires safety. In modern cultures, road accidents are a major source of death and serious injuries. Road traffic injuries are a substantial yet underserved public health issue around the world that requires immediate attention. Conservative preventive approaches to successful long‐term prevention are needed. Road transportation is the most complex and deadly system that people must face on a daily basis. Road safety management aims to preserve and improve the current safety of a road network by minimizing collisions and creating a safe road environment for its users, allowing it to be used in an effective and safe way in the future. It is concerned with the execution of road safety regulations, administration, and organization in the authorities in charge of reducing road collisions and fatalities.

## AUTHOR CONTRIBUTIONS

**Sirwan K. Ahmed**: Conceptualization; data curation; formal analysis; investigation; methodology; project administration; resources; software; supervision; validation; visualization; writing—original draft; writing—review and editing. **Mona G. Mohammed**: Data curation; methodology; resources; software; validation; visualization; writing—original draft; writing—review and editing. **Salar O. Abdulqadir**: Data curation; formal analysis; investigation; methodology; resources; validation; visualization; writing—review and editing. **Rabab G. Abd El‐Kader**: Data curation; investigation; resources; validation; writing—review and editing. **Nahed A. El‐Shall**: Resources; writing—review and editing. **Deepak Chandran**: Data curation; formal analysis; resources; software; validation; visualization; writing—review and editing. **Mohammad E. Ur Rehman**: Resources; writing—review and editing. **Kuldeep Dhama**: Data curation; formal analysis; investigation; methodology; resources; software; supervision; validation; visualization; writing—review and editing.

## CONFLICT OF INTEREST STATEMENT

The authors declare no conflict of interest.

## ETHICS STATEMENT

Not required.

## TRANSPARENCY STATEMENT

The lead author Sirwan Khalid Ahmed affirms that this manuscript is an honest, accurate, and transparent account of the study being reported; that no important aspects of the study have been omitted; and that any discrepancies from the study as planned (and, if relevant, registered) have been explained.

## Data Availability

All data presented in the present review is available online and can be accessed from the appropriate reference in the reference list.
